# Desired support system to eradicate urban homelessness: an exploratory descriptive study

**DOI:** 10.12688/f1000research.73536.1

**Published:** 2022-01-13

**Authors:** Noor Ashikin Mohd Rom, Nurbani Md. Hassan, Al-Mansor Abu Said, Burhanuddin Bachik

**Affiliations:** 1 Faculty of Management, Multimedia University, Cyberjaya, Selangor, 63100, Malaysia; 2 Faculty of Business, Multimedia University, Cyberjaya, Selangor, 63100, Malaysia; 3 Kementerian Pembangunan Wanita, Keluarga dan Masyarakat, Bandar Melaka, Melaka, 75564, Malaysia

**Keywords:** Homeless, vagrants, social-ecological model, government policy, support system

## Abstract

Background - The new increasing homeless lately consist of women, children, youth, the elderly and marginalized ethnic or migrant groups. Some of them are working and earn salaries, however, the income is not sufficient to live modestly.

Purpose – The purpose of this research is to establish a desired support system to eradicate urban homelessness in the country.

Design/methodology/approach – This is an exploratory descriptive method study which employed quantitative techniques.  The study employed a social ecological model to investigate behavior of homeless via multiple levels of influences including intrapersonal, interpersonal, organizational, community and public policy. Surveys have been conducted on sixty-five homeless individuals.

Findings – It was found that support systems and structures should be derived from the whole streams from families, communities, organizations and government. Employment opportunities, long-term shelters within the community places and highly demanded skills are needed to improve their living condition.

Research limitations – This study is only focused on the socio-economic structures of the homeless in a capital city.

Originality/value – This is an empirical research using a social ecological model for the homeless in the Kuala Lumpur area. Research on homeless study has received little attention and has yet to be fully explored.

## Introduction

According to the Malaysian National Key Result Area (NKRA), every citizen should have access to necessities, including a home. Homeless people are often portrayed as causing inconvenience and annoyance to the public. The living conditions of the homeless people and surrounding environments may tarnish the image of the capital city, Kuala Lumpur. It was reported that homeless people have not been fully accepted by the authorities and are perceived as a public nuisance. ^1^

The welfare department, Jabatan Kebajikan Masyarakat (JKM) reported approximately 2,472 homeless people comprising 3,117 adults and 355 children. In terms of ethnicity, 48.9% are Malay, 15.6% are Indian, 13.4% are Chinese, and 1.8% are other ethnicities. Nonetheless, 20.4% of homeless people are non-Malaysians. The Malaysian government has established the Desa Bina Diri (DBD) for homeless and impoverished people. The DBD offers protection, provides rehabilitation, and equips them with essential skills to be productive, independent, secure a job, and adapt to the community. In addition, homeless individuals can join DBD voluntarily or by court order following the provisions of the Destitute Persons Act 1977. ^1^

The number of homeless people is increasing due to the global economic uncertainty of the COVID-19 pandemic worldwide. This phenomenon is worrying and should not only be the responsibility of policymakers, but the whole structure consisting of the family, society, and organisations to collaborate to curb the desperate living conditions of unfortunate individuals. Therefore, this study aims to establish a desired support system to eradicate urban homelessness in the country.

## Related works

A home is a place that provides shelter, privacy, warmth, love, relaxation, health, happiness, stability, and paradise ^2^ and is essential for the wellbeing of man. ^3^^,^^4^ Economic uncertainty and the increased living costs contribute to the increase of homeless people in Malaysia. ^5^ One common perception is that homeless people worldwide are deprived of fundamental human needs and are often poor and financially insecure. ^5^^–^^7^ Homeless' lives becoming worsen due to lack of family and social support. They felt abandoned and lost hope. ^8^

Housing costs and food prices have been increasing rapidly over the years, leading to an increase in beggars and vagrants. ^9^ The cause of homelessness varies and is linked to unemployment, low income, unable to afford a proper house, lack of affordable housing, and unavailability of transport to work. ^7^^,^^9^ In addition, the unemployment issue among the homeless can be prevented through training and equipping them with essential skills, including reading, grooming, and job interview skills. ^6^^,^^10^^,^^11^ Policymakers should comprehend the various perspectives that influence homelessness, such as the socio-economic systems, inadequate housing, and inequitable welfare that have dire consequences for families and individuals. ^7^^,^^10^

The Social Ecological Model describes that the behaviour of individuals is influenced and encouraged by their surroundings. There are five influence levels: intrapersonal, interpersonal, organisational, community, and public policy. ^12^^,^^13^ Homelessness is influenced by circumstances, socio-economy, and environmental conditions. ^13^^,^^14^ A framework that incorporates the five crucial levels of the Social Ecological Model has been established. The first level consists of the individual’s demography and economic status, and the second level is the interpersonal relationship with family, peers and network. Additionally, the third level consists of the relationship between the individual and the community, including associations and informal networks. The fourth level is organisations that deal with rules and regulations, and the fifth level is policy which comprises local, state, and national policies for citizens. ^15^

## Methods

This study employed an exploratory descriptive study using the Statistical Package for the Social Sciences (SPSS Statistics 26) to investigate the behaviour of homeless via multiple influences, such as intrapersonal, interpersonal, organisational, community, and public policy. The study employed a social ecological Model to survey sixty-five (65) homeless people. The survey respondents were identified from soup kitchens organised by numerous non-governmental organisations (NGOs) around Kuala Lumpur.

The interviews were conducted at the respondent’s location with their full consent. The Ethical Approval No.: EA1042021 was obtained from Technology Transfer Office (TTO), Multimedia University, to conduct this study. Prior to the interview session, the objectives of this study and respondents’ consent to conduct face-to-face interviews for the data collection were obtained. All 65 respondents had given their verbal consent to be interviewed. The questionnaires designed included close-ended questions with a 5-Point Likert scale. The survey questionnaire was divided into five sections, namely section (1) individual, (2) interpersonal, (3) community, (4) organisational and (5) policy. Section 1 aimed to collect demographic information of respondents, reasons for homelessness and challenges faced. Section 2 included relationships with families and peers, while Section 3 determined relationships in the community. Moreover, Section 4 collected information on the aid received from various organisations and Section 5 focused on the respondent’s perception of the government’s policies towards them.

### Findings

The findings on the demography of the respondents are illustrated in Table 1 below.

**Table 1.** Demographic of respondents.

	Frequency	Percentage
Gender		
Male	47	72.3
Female	18	27.7
Ethnicity		
Malay	45	69.2
Chinese	9	13.8
Indian	11	16.9
Age (Years Old)		
18-30	3	4.6
31-50	29	44.7
51-70	19	29.2
> 70	14	21.5
Marital status		
Single	30	46.2
Married	5	7.7
Divorce	27	41.5
Widowed	3	4.6
Education		
Primary and Secondary	44	67.7
STPM/Diploma/Certificate	18	27.7
None	3	4.6
Duration of vagrancy		
< 5 years	14	21.5
6-10 years	21	32.3
11-15 years	6	9.2
16-20 years	9	13.8
21-30 years	4	6.2
> 30 years	11	16.9
Current economic activity		
Working (monthly salary)	11	16.9
Daily income	15	23.1
Unemployed	29	44.6
Begging	10	15.4

The crosstab analysis indicates that single, Malay males represent the highest number of homeless individuals staying on the streets in Kuala Lumpur. Divorcees are the second-highest individuals that had to leave their houses to live on their own. The majority of respondents were between 18-50 years old. Conversely, there are around 33(50.7%) individuals who are more than 50 years old. In addition, 67.7% possess basic education, and 27.7% have higher qualifications. Nevertheless, most of the respondents are computer illiterate. Approximately 78% of respondents have been living on the streets for more than five years. Eleven respondents who were above 70 years old have been homeless for more than 30 years. Most of the respondents below 60 years old claimed that they needed a job to survive, while those above 60 years old were unaware of how to generate income. Lastly, 10 (15.4%) respondents admitted earning their living by begging.

## Discussion

Based on the mean score results shown in Table 2, the respondents became homeless due to loss of jobs, family breakdown, and the inability to afford accommodation. Most of the respondents are in dire need of shelter and wish to live a conventional life. Conversely, several respondents were forced to live on the street due to unforeseen circumstances; however, some chose to live on the streets as it is easier for them to commute. The low mean scores indicate that the respondents are not computer literate, unskilled, and lack administration, marketing or business knowledge. Nevertheless, they are inclined towards service jobs, such as cleaning, shop assistants, selling tins, and other odd jobs.

**Table 2.** Mean and standard deviation.

Section	Measures of the constructs	Mean	Std. Dev.
Individual	Unemployment/Loss of Income	4.5231	1.25135
	I became homeless by choice	2.7538	1.69587
	Family Breakdown	3.5077	1.56248
	Lack of affordable place	4.5385	.90272
	Basic computer literacy	2.5077	1.77767
	Intermediate computer	1.7692	1.49759
	Administration/Marketing	2.1231	1.51562
	Services	4.2308	1.49759
	Business knowledge	1.1846	.72656
Interpersonal	Have a good relationship with family	1.4462	.79118
	I keep in touch with friends, families or relatives	1.7692	.96451
	Do not aware of family matters	3.1077	.83147
	I have plans for future	3.3538	1.02211
	I can get out from this situation in future	3.4000	1.23491
Community	Going to worship places or other community places	1.9077	3.38528
	Have an opportunity for involvement in community activities	1.6154	1.04122
	Felt uncared and alone	2.4000	1.22219
	Feel insecure to join the community	3.4769	1.44814
	Not rely on others	1.8923	.97023
Organization	I received food assistance	4.8154	.84580
	I received one-off monetary assistance	3.2615	1.91439
	I received regularly monetary assistance	1.0462	.21145
	I received donations other than food (sanitary, etc)	4.7538	.96874
	I am happy staying at Desa Bina Diri (DBD)	1.7538	1.26282
Policy	I do not like to be caught by government agency/KLCH (DBKL)	4.6615	.56670
	Government should leave us alone	2.2154	1.12489
	Government agencies are friendly towards homeless	3.5538	.81069
	Current policies did not favour us	2.8615	.74743

The low mean score and standard deviation score for the interpersonal section illustrate that most respondents do not have a good relationship with their families. Most of the respondents are unaware of their children’s progress and have not kept in touch with their families in a long time. In addition, respondents do not want to reunite with their families, have lost contact with friends, and live in their circle. Nonetheless, they claimed to have future plans and wished to free themselves from this situation as soon as possible.

The low mean score for the community section indicates that homeless people lack of community relationships. Nonetheless, the high standard deviation result demonstrates that several respondents visit places of worship and other community spaces. The low community involvement indicates that they prefer to interact within their small selected circle of homeless peers. Moreover, they hardly participate in community activities and are insecure about interacting with outsiders. Nevertheless, the respondents acknowledged that they required assistance for food and other necessities from others.

The high mean scores for the organisation section indicate that respondents are overwhelmed with the aids provided by non-governmental organisations (NGOs) such as food, sanitary products, and one-off monetary assistance from the religious office, Majlis Agama Islam Wilayah Persekutuan (MAIWP). Conversely, respondents are not happy to stay in Desa Bina Diri (DBD), a shelter that offers protection, provides rehabilitation, and equips them with essential skills to be productive, independent, secure a job, and adapt to the community. The high mean scores for policy denotes that the respondents do not like to be apprehended by government agencies such as Kuala Lumpur City Hall, also known as Dewan Bandaraya Kuala Lumpur (DBKL) and placed at DBD. Additionally, the respondents claimed that the current governmental policies do not favour them. Alternately, respondents perceived these government agencies as friendly and helpful.

## Conclusion

Homeless people require physical assistance (shelter, food, and medication) and spiritual nurture to alter their mindset and behaviour. They have to belong to the community and should have a circle of positive peers. This support system must include family, community, and organisations (government agencies and NGOs). In addition, rulers should collaborate and be responsive to homelessness issues. Every stage of these structures must collaborate to provide resources and manage the vagrancy or homeless matter.

Family, peers, and relatives are the closest support structure for unfortunate individuals. In addition, the society or communities near these impoverished individuals are vital pillars to ensure no one is left hungry and homeless. There are growing numbers of NGOs that provide immediate support to homeless regarding food, sanitary products, and clothes. Policymakers have also devised various programs to tackle homelessness, such as establishing DBD, Anjung Singgah (temporary shelter) and providing job opportunities for the homeless. Nevertheless, the number of homeless individuals is not reducing. Consequently, policymakers must provide long-term shelters or houses to eradicate urban homelessness. Necessities such as accommodation are vital for homeless people to restart their lives and quickly adapt to the surrounding community.

## Author contributions

First author conceptualized the research idea, theoretical framework and write the paper. Second author revised the article; fourth visualized and third author reviewed the article for submission.

## Competing interest

The authors agree that this research was conducted in the absence of any self-benefits, commercial or financial conflicts and declare absence of conflicting interests with the funders.

## Data availability

Figshare. Desired Support System to Eradicate Urban Homelessness: An Exploratory Descriptive Study. DOI: https://doi.org/10.6084/m9.figshare.16529391.v1 ^16^

Data are available under the terms of the Creative Commons Zero “No rights reserved” data waiver (CC BY 4.0 Public domain dedication).

## Grant information

A Fundamental Research Grant Scheme (FRGS) FRGS/1/2019/SS06/MMU/03/1 was awarded to the researchers by Ministry of Higher Education, Malaysia in 2019. We confirm that the funders had no role in study design, data collection and analysis, decision to publish, or preparation of the manuscript.

## Ethics approval and consent to participate

Ethical Approval No.: EA1042021 for Homeless project was obtained from Technology Transfer Office (TTO), Multimedia University. Respondents have given verbal consent to publish.
