# Desired support system to eradicate urban homelessness: an exploratory descriptive study

**DOI:** 10.12688/f1000research.73536.2

**Published:** 2024-09-16

**Authors:** Noor Ashikin Mohd Rom, Nurbani Md. Hassan, Al-Mansor Abu Said, Burhanuddin Bachik

**Affiliations:** 1 Faculty of Management, Multimedia University, Cyberjaya, Selangor, 63100, Malaysia; 2 Faculty of Business, Multimedia University, Cyberjaya, Selangor, 63100, Malaysia; 3 Kementerian Pembangunan Wanita, Keluarga dan Masyarakat, Bandar Melaka, Melaka, 75564, Malaysia

**Keywords:** Homeless, vagrants, social-ecological model, government policy, support system

## Abstract

Background - The new increasing homeless lately consist of women, children, youth, the elderly and marginalized ethnic or migrant groups. Some of them are working and earn salaries, however, the income is not sufficient to live modestly.

Purpose – The purpose of this research is to establish a desired support system to eradicate urban homelessness in the country.

Design/methodology/approach – This is an exploratory descriptive method study which employed quantitative techniques.  The study employed a social ecological model to investigate behavior of homeless via multiple levels of influences including intrapersonal, interpersonal, organizational, community and public policy. Surveys have been conducted on sixty-five homeless individuals.

Findings – It was found that support systems and structures should be derived from the whole streams from families, communities, organizations and government. Employment opportunities, long-term shelters within the community places and highly demanded skills are needed to improve their living condition.

Research limitations – This study is only focused on the socio-economic structures of the homeless in a capital city.

Originality/value – This is an empirical research using a social ecological model for the homeless in the Kuala Lumpur area. Research on homeless study has received little attention and has yet to be fully explored.

## Introduction

According to the Malaysian National Key Result Area (NKRA), every citizen should have access to necessities, including a home. Homeless people are often portrayed as causing inconvenience and annoyance to the public. The living conditions of the homeless people and surrounding environments may tarnish the image of the capital city, Kuala Lumpur. It was reported that homeless people have not been fully accepted by the authorities and are perceived as a public nuisance. ^1^

The Malaysian Department of Social Welfare ( *Jabatan Kebajikan Masyarakat*) reported approximately 2,472 homeless people comprising 3,117 adults and 355 children. In terms of ethnicity, 48.9% are Malay, 15.6% are Indian, 13.4% are Chinese, and 1.8% are other ethnicities. Nonetheless, 20.4% of homeless people are non-Malaysians. The Malaysian government has established the *Desa Bina Diri (DBD)* for homeless and impoverished people. The DBD offers protection, provides rehabilitation, and equips them with essential skills to be productive, independent, secure a job, and adapt to the community. In addition, homeless individuals can join DBD voluntarily or by court order following the provisions of the Destitute Persons Act 1977. ^1^

The number of homeless people is increasing due to the global economic uncertainty of the COVID-19 pandemic worldwide. This phenomenon is worrying and should not only be the responsibility of policymakers, but the whole structure consisting of the family, society, and organisations to collaborate to curb the desperate living conditions of unfortunate individuals. Therefore, this study aims to establish a desired support system to eradicate urban homelessness in the country.

## Related works

A home is a place that provides shelter, privacy, warmth, love, relaxation, health, happiness, stability, and paradise ^2^ and is essential for the wellbeing of man. ^3^^,^^4^ Economic uncertainty and the increased living costs contribute to the increase of homeless people in Malaysia. ^5^ One common perception is that homeless people worldwide are deprived of fundamental human needs and are often poor and financially insecure. ^5^^–^^7^ Homeless’ lives becoming worsen due to lack of family and social support. ^8^ They felt abandoned and lost hope. ^9^ Housing costs and food prices have been increasing rapidly over the years, leading to an increase in beggars and vagrants. ^10^ The cause of homelessness varies and is linked to unemployment, low income, unable to afford a proper house, lack of affordable housing, and unavailability of transport to work. ^7^^,^^9^ Nevertheless, Policymakers should understand the diverse factors influencing homelessness, including socio-economic systems, inadequate housing, and inequitable welfare, as these issues have severe consequences for families and individuals. ^7^^,^^10^ Homelessness eradication program must focus on providing stable housing to reduce stress and improve well-being, particularly in quality neighbourhoods that foster social connections. ^9^

The unemployment issue among the homeless can be prevented through training and equipping them with essential skills, including reading, grooming, and job interview skills. ^6^^,^^11^^,^^12^ It was suggested that developing occupational skills can positively impact the well-being and outcomes of those experiencing unemployment and homelessness. ^13^^–^^15^ It was also noted that while homeless individuals can improve and able to plan for better future with proper training and rehabilitation, success depends on their determination to change. ^16^^,^^17^ Surprisingly there was 17.8% of those who underwent rehabilitation, or eight out of 45, returned to the streets. ^18^

The concept of community is inherently complex, often defined by the specific and narrow scope of interpersonal relationships among residents in a particular area. ^19^ When individuals feel disconnected from society and unable to participate in valued activities, it can lead to reduced social trust and cohesion, particularly as people experience vastly different living conditions. ^20^^,^^21^ People experiencing homelessness are especially vulnerable to intense social stigma and are often marginalized. ^21^ Despite their isolation, homeless individuals still hope for basic physical assistance, such as shelter, food, and medical care, along with societal support, meaningful connections with caregivers, and integrated care programs. ^22^^,^^23^ Homelessness prevention should be a collaborative effort involving all levels of government and various institutions beyond the homelessness sector. Structural and systems prevention need active participation from mainstream systems, while prevention methods within the homelessness sector, such as early intervention, housing stabilization and eviction prevention, work best when integrated with these broader systems. ^24^ Neoliberal policies reinforce deep-rooted systems of care and control, influencing Malaysia’s homelessness policy through a mix of punitive and supportive state measures. Rights and protections should not be nullified simply because someone becomes ‘destitute’. ^25^

The Social Ecological Model describes that the behaviour of individuals is influenced and encouraged by their surroundings. There are five influence levels: individual/intrapersonal, interpersonal, organisational, community, and public policy. ^26^^,^^27^ Homelessness is influenced by circumstances, socio-economy, and environmental conditions. ^27^^,^^28^ A framework that incorporates the five crucial levels of the Social Ecological Model has been established. The first level consists of the individual’s demography and economic status, and the second level is the interpersonal relationship with family, peers and network. Additionally, the third level consists of the relationship between the individual and the community, including associations and informal networks. The fourth level is organisations that deal with rules and regulations, and the fifth level is policy which comprises local, state, and national policies for citizens. ^29^

## Methods

This study employed an exploratory descriptive study using the Statistical Package for the Social Sciences (SPSS Statistics 26) to investigate the behaviour of homeless via multiple influences, such as intrapersonal, interpersonal, organisational, community, and public policy. Convenience sampling was applied in this study, as this method involves selecting individuals who are readily available and willing to participate. The study employed a social ecological Model to survey sixty-five (65) homeless people. The survey respondents were identified by *Pertubuhan Kebajikan ArRiqab*, one of the active non-governmental organisations (NGOs) that assisting homeless people in Kuala Lumpur area. A simple regression analysis requires a minimum of 50 samples and sample-to-variable ratio suggests a minimum observation-to-variable ratio of 5:1. ^30^ Additionally, minimum sample size of N ≥ 50+m (where m is the number of predictors) was recommended. ^31^ The minimum sample size by applying ^31^ method is 55, however, the researchers managed to get 65 respondents.

The interviews were conducted at the respondents’ location with their full consent. The Ethical Approval No.: EA1042021 was obtained from Technology Transfer Office (TTO), Multimedia University, to conduct this study. Before the survey session, the objectives of the study were explained, and respondents’ consent for data collection was obtained. All 65 respondents provided their verbal consent to participate in the survey. This is a structured interview using questionnaires. The questionnaires designed included close-ended questions with a 5-Point Likert scale. The survey questionnaire was divided into five sections, namely section (1) individual, (2) interpersonal, (3) community, (4) organisational and (5) policy. Section 1 aimed to collect demographic information of respondents, reasons for homelessness and challenges faced. Section 2 included relationships with families and peers, while Section 3 determined relationships in the community. Moreover, Section 4 collected information on the aid received from various organisations and Section 5 focused on the respondent’s perception of the government’s policies towards them.

### Findings

The findings on the demography of the respondents are illustrated in Table 1 below.

**Table 1.** Demographic of respondents.

	Frequency	Percentage
Gender		
Male	47	72.3
Female	18	27.7
Ethnicity		
Malay	45	69.2
Chinese	9	13.8
Indian	11	16.9
Age (Years Old)		
18-30	3	4.6
31-50	29	44.7
51-70	19	29.2
> 70	14	21.5
Marital status		
Single	30	46.2
Married	5	7.7
Divorce	27	41.5
Widowed	3	4.6
Education		
Primary and Secondary	44	67.7
STPM/Diploma/Certificate	18	27.7
None	3	4.6
Duration of vagrancy		
< 5 years	14	21.5
6-10 years	21	32.3
11-15 years	6	9.2
16-20 years	9	13.8
21-30 years	4	6.2
> 30 years	11	16.9
Current economic activity		
Working (monthly salary)	11	16.9
Daily income	15	23.1
Unemployed	29	44.6
Begging	10	15.4

The crosstab analysis indicates that single, Malay males represent the highest number of homeless individuals staying on the streets in Kuala Lumpur. Divorcees are the second-highest individuals that had to leave their houses to live on their own. The majority of respondents were between 18-50 years old. Conversely, there are around 33(50.7%) individuals who are more than 50 years old. In addition, 67.7% possess basic education, and 27.7% have higher qualifications. Nevertheless, most of the respondents are computer illiterate. Approximately 78% of respondents have been living on the streets for more than five years. Eleven respondents who were above 70 years old have been homeless for more than 30 years. Most of the respondents below 60 years old claimed that they needed a job to survive, while those above 60 years old were unaware of how to generate income. Lastly, 10 (15.4%) respondents admitted earning their living by begging.

Based on the mean score results shown in Table 2, the respondents became homeless due to loss of jobs, family breakdown, and the inability to afford accommodation. Most of the respondents are in dire need of shelter and wish to live a conventional life. Conversely, several respondents were forced to live on the street due to unforeseen circumstances; however, some chose to live on the streets as it is easier for them to commute. The low mean scores indicate that the respondents are not computer literate, unskilled, and lack administration, marketing or business knowledge. Nevertheless, they are inclined towards service jobs, such as cleaning, shop assistants, selling tins, and other odd jobs.

**Table 2.** Mean and standard deviation.

Section	Measures of the constructs	Mean	Std. Dev.
Individual	Unemployment/Loss of Income	4.5231	1.25135
	I became homeless by choice	2.7538	1.69587
	Family Breakdown	3.5077	1.56248
	Lack of affordable place	4.5385	.90272
	Basic computer literacy	2.5077	1.77767
	Intermediate computer	1.7692	1.49759
	Administration/Marketing	2.1231	1.51562
	Services	4.2308	1.49759
	Business knowledge	1.1846	.72656
Interpersonal	Have a good relationship with family	1.4462	.79118
	I keep in touch with friends, families or relatives	1.7692	.96451
	Do not aware of family matters	3.1077	.83147
	I have plans for future	3.3538	1.02211
	I can get out from this situation in future	3.4000	1.23491
Community	Going to worship places or other community places	1.9077	3.38528
	Have an opportunity for involvement in community activities	1.6154	1.04122
	Felt uncared and alone	2.4000	1.22219
	Feel insecure to join the community	3.4769	1.44814
	Not rely on others	1.8923	.97023
Organization	I received food assistance	4.8154	.84580
	I received one-off monetary assistance	3.2615	1.91439
	I received regularly monetary assistance	1.0462	.21145
	I received donations other than food (sanitary, etc)	4.7538	.96874
	I am happy staying at *Desa Bina Diri (DBD)*	1.7538	1.26282
Policy	I do not like to be caught by government agency/KLCH ( *DBKL*)	4.6615	.56670
	Government should leave us alone	2.2154	1.12489
	Government agencies are friendly towards homeless	3.5538	.81069
	Current policies did not favour us	2.8615	.74743

The low mean score and standard deviation score for the interpersonal section illustrate that most respondents do not have a good relationship with their families. Most of the respondents are unaware of their children’s progress and have not kept in touch with their families in a long time. In addition, respondents do not want to reunite with their families, have lost contact with friends, and live in their circle. Nonetheless, they claimed to have future plans and wished to free themselves from this situation as soon as possible.

The low mean score for the community section indicates that homeless people lack of community relationships. Nonetheless, the high standard deviation result demonstrates that several respondents visit places of worship and other community spaces. The low community involvement indicates that they prefer to interact within their small selected circle of homeless peers. Moreover, they hardly participate in community activities and are insecure about interacting with outsiders. Nevertheless, the respondents acknowledged that they required assistance for food and other necessities from others.

The high mean scores for the organisation section indicate that respondents are overwhelmed with the aids provided by non-governmental organisations (NGOs) such as food, sanitary products, and one-off monetary assistance from the religious office, *Majlis Agama Islam Wilayah Persekutuan (MAIWP)*. Conversely, respondents are not happy to stay in *Desa Bina Diri (DBD)*, a shelter that offers protection, provides rehabilitation, and equips them with essential skills to be productive, independent, secure a job, and adapt to the community. The high mean scores for policy denotes that the respondents do not like to be apprehended by government agencies such as Kuala Lumpur City Hall, also known as *Dewan Bandaraya Kuala Lumpur (DBKL) *and placed at DBD. Additionally, the respondents claimed that the current governmental policies do not favour them. Alternately, respondents perceived these government agencies as friendly and helpful.

## Discussion

The first level of the Social Ecological Model highlights how individual demographics and economic status play a crucial role in homelessness. It shows that many homeless individuals in Malaysia struggle with insufficient income, inability to afford housing, and a lack of skills. Previous studies have identified economic instability and rising living costs as significant contributors to the growing homeless population in the country. ^5^ These findings are further supported by Refs. 5, 10 linking homelessness to factors such as unemployment, low income, inability to secure proper housing, lack of affordable housing, and limited access to transportation for work. ^7^^,^^10^ Compounding these challenges, many homeless individuals experience a sense of abandonment due to fractured family relationships. ^8^ The study also revealed that homeless respondents lacked the essential skills needed to obtain stable employment. This observation aligns with the research of Refs. 14, 15 which demonstrated that developing occupational skills can significantly enhance the well-being and prospects of those facing unemployment and homelessness. The persistent lack of education, training, and life skills continues to be a major barrier to employment. ^13^^,^^32^

The second level of the Social Ecological Model emphasizes that homeless individuals frequently suffer from weak interpersonal relationships with family, peers, and social networks. Many respondents reported experiencing family conflicts and strained ties with relatives and friends. This observation is consistent with previous studies ^5^^,^^7^^,^^9^ which indicate that homeless individuals often feel abandoned, endure social isolation, and lack sufficient social support and resources. It was revealed that homeless individuals suffer from deficiencies in familial and community support, as well as limited interactions with service providers. ^9^ Nevertheless, the finding revealed that some respondents expressed confidence in their ability to overcome their current situation. The finding is consistent with ^17^ who found that the homeless who often viewed as a burden on society due to their nonconformity to social norms, can transform themselves when given proper training and rehabilitation, as they possess the ability to plan for a better future. However, these efforts are unlikely to succeed if the homeless lack the determination to resolve their issues, causing them to revert to their old ways, even after receiving support ^16^^,^^18^ discovered that about eight out of 45, returned to the streets despite undergoing rehabilitation programs.

The third level focuses on the relationship between homeless individuals and the broader community, including associations and informal networks, revealing that they often feel insecure and marginalized from societal participation due to their circumstances. This finding aligns with the study by Ref. 9 who observed that homeless individuals frequently experience isolation, remain silent, and perceive discrimination because of their situation ^.^ Similarly, it was noted that perceived estrangement and social exclusion can exacerbate societal divides, diminishing social trust and cohesion, and leaving individuals feeling disconnected and unable to engage in meaningful activities. ^20^^,^^21^

The fourth level of the model focuses on the role of organizations in impacting the lives of homeless individuals. These organizations provide essential support, such as food, monetary assistance, and shelter. This finding aligns with ^22^^,^^23^ who emphasized that homeless individuals require physical support in the form of shelter, food, and medical care, as well as societal interventions to address fundamental human needs. Supportive relationships with caregivers and targeted programs that integrate care and services are crucial approaches to consider in effectively assisting the homeless.

The fifth level is policy which comprises local, state, and national policies for citizens. The respondents claimed that the current governmental policies do not favour them and should not interfere with their life. The findings align with the study conducted by Ref. 25 stated that the authorities under Destitute Persons Act 1977 (DPA 1977) can forcibly remove individuals they consider homeless and place them in *Desa Bina Diri (DBD),* a shelter that offers protection with the court order. DPA 1977 has faced significant criticism for violating personal liberty and freedom of movement of person who is categorized as a destitute person. Nonetheless, the finding also show that some respondents viewed these government agencies as friendly and supportive. The finding corroborates with ^25^^,^^33^ who revealed that some homeless individuals, particularly senior citizens, were rescued from their difficult lives on the streets by the authorities. They were given necessary assistance including shelter. Nevertheless, the concept of ‘rehabilitation’ must be critically examined to determine whether it genuinely addresses the needs of those experiencing homelessness. ^25^ The policies should focus on establishing a robust support system for the homeless, while also empowering the elderly and strengthening their communities. ^33^

## Conclusion

To conclude, eradicating urban homelessness requires a comprehensive approach rooted in the five pillars of the structural ecological model. This study underscores the importance of equipping homeless individuals with employment skills (individual), fostering peer support (interpersonal), promoting social acceptance (community), ensuring access to rehabilitation services (organizational), and implementing a coordinated governmental strategy (policy). While current efforts provide essential support like health services, temporary shelters, and food, they often fail to address the other root causes of homelessness, such as unemployment, loss of income, lack of skills and social support. Therefore, it is imperative for the government to intensify efforts by funding programs that enhance literacy and provide Technical and Vocational Education and Training (TVET). These programs should offer formal, non-formal, and informal learning opportunities that may collaborate with communities and organizations that empower individuals to secure meaningful employment and rebuild their lives. Additionally, the government shall prioritize accessible housing for the homeless, similar to the “Projek Perumahan Rakyat Termiskin (PPRT)” or Housing Project for the Absolute Poor, ensuring that these projects are widespread and located near essential amenities. Providing permanent housing to meet the basic needs of the homeless can significantly alleviate their hardships and improve their overall well-being. By providing stable housing, communities, and organizations can further support the homeless by offering employment opportunities, with the government incentivizing employers to participate. This integrated approach is vital to enabling homeless individuals to reintegrate into society and sustain long-term stability.

A comprehensive and well-structured support system is essential to effectively eradicate urban homelessness in the country. This system should be designed to address the root causes of homelessness, such as poverty, lack of affordable housing, unemployment and lack of social support. It would involve coordinated efforts from government agencies, non-governmental organizations, and the private sector, focusing on providing not only immediate relief but also long-term solutions. These solutions could include affordable housing programs, job training and employment opportunities, and social support networks. By integrating these components into a cohesive and sustainable framework, the desired support system would not only provide immediate assistance to those experiencing homelessness but also work towards preventing future homelessness, ultimately leading to the complete eradication of urban homelessness in the country.

## Author contributions

First author conceptualized the research idea, theoretical framework and write the paper. Second author revised the article; fourth visualized and third author reviewed the article for submission.

## Data availability

Figshare. Desired Support System to Eradicate Urban Homelessness: An Exploratory Descriptive Study. DOI: https://doi.org/10.6084/m9.figshare.16529391.v1 ^34^

Data are available under the terms of the Creative Commons Zero “No rights reserved” data waiver (CC BY 4.0 Public domain dedication).

## Ethics approval and consent to participate

Ethical Approval No.: EA1042021 for Homeless project was obtained from Technology Transfer Office (TTO), Multimedia University. Respondents have given verbal consent to publish.
