# Supplementary material for: Desired support system to eradicate urban homelessness: an exploratory descriptive study
Source: F1000Res. 2024 Sep 16;11:41. Originally published 2022 Jan 13. [Version 2] doi: 10.12688/f1000research.73536.2 (PMC11425782; doi:10.12688/f1000research.73536.2)
Supplement: Peer review history

# Reviewer report: Reviewer response for version 2

**DOI:** 10.5256/f1000research.171283.r324087

**Published:** 2024-09-25

**Authors:** K. Rewathy

**Affiliations:** 1 Lecturer in Geography, University of Colombo, Colombo, Western Province, Sri Lanka

The article was well revised and no further comments to make.

Is the work clearly and accurately presented and does it cite the current literature?

Partly

If applicable, is the statistical analysis and its interpretation appropriate?

I cannot comment. A qualified statistician is required.

Are all the source data underlying the results available to ensure full reproducibility?

Yes

Is the study design appropriate and is the work technically sound?

Yes

Are the conclusions drawn adequately supported by the results?

No

Are sufficient details of methods and analysis provided to allow replication by others?

Yes

Reviewer Expertise:

Social Geography, Human geography, GIS and Remote sensing, Urban Geography, Environmental studies

I confirm that I have read this submission and believe that I have an appropriate level of expertise to confirm that it is of an acceptable scientific standard.

# Reviewer report: Reviewer response for version 2

**DOI:** 10.5256/f1000research.171283.r324088

**Published:** 2024-09-18

**Authors:** Zakiyah Jamaluddin

**Affiliations:** 1 School of Applied Psychology, Social Work & Policy, Universiti Utara Malaysia (UUM), Kuala Lumpur, Malaysia

I have read all the revised statements and information and I accept the correction being made by the authors.

Is the work clearly and accurately presented and does it cite the current literature?

Yes

If applicable, is the statistical analysis and its interpretation appropriate?

Yes

Are all the source data underlying the results available to ensure full reproducibility?

Partly

Is the study design appropriate and is the work technically sound?

Yes

Are the conclusions drawn adequately supported by the results?

Partly

Are sufficient details of methods and analysis provided to allow replication by others?

Partly

Reviewer Expertise:

NA

I confirm that I have read this submission and believe that I have an appropriate level of expertise to confirm that it is of an acceptable scientific standard.

# Reviewer report: Reviewer response for version 1

**DOI:** 10.5256/f1000research.77191.r169771

**Published:** 2023-05-09

**Authors:** K. Rewathy

**Affiliations:** 1 Lecturer in Geography, University of Colombo, Colombo, Western Province, Sri Lanka

The paper is well-written, it addresses the major issues of homelessness in Malaysia and it tries to identify a suitable system to eradicate the homelessness to some extent. It addresses a timely important topic. However, there are a few instances where it may be improved:

- Italicize any local terms that are utilized to identify the term clearly.
- The literature section should be expanded with relevant content that highlights homelessness eradication approaches.
- For the justification, previous research must be acknowledged in the discussion section.
- The findings must back up the conclusion, rather than more general.

Is the work clearly and accurately presented and does it cite the current literature?

Partly

If applicable, is the statistical analysis and its interpretation appropriate?

I cannot comment. A qualified statistician is required.

Are all the source data underlying the results available to ensure full reproducibility?

Yes

Is the study design appropriate and is the work technically sound?

Yes

Are the conclusions drawn adequately supported by the results?

No

Are sufficient details of methods and analysis provided to allow replication by others?

Yes

Reviewer Expertise:

Social Geography, Human geography, GIS and Remote sensing, Urban Geography, Environmental studies

I confirm that I have read this submission and believe that I have an appropriate level of expertise to confirm that it is of an acceptable scientific standard, however I have significant reservations, as outlined above.

# Response

**Published:** 2024-08-31

**Authors:** Noor Ashikin Mohd Rom

**Affiliations:** Multimedia University, Malaysia

1. All local terms in Italic font.

 2. The literature review on approaches to eradicating homelessness has been updated accordingly.

^9^Nevertheless, policymakers should understand the diverse factors influencing homelessness, including socio-economic systems, inadequate housing, and inequitable welfare, as these issues have severe consequences for families and individuals. ^7^^,^^10^ Homelessness eradication program must focus on providing stable housing to reduce stress and improve well-being, particularly in quality neighbourhoods that foster social connections ^9^. It was suggested that developing occupational skills can positively impact the well-being and outcomes of those experiencing unemployment and homelessness. ^13,14,15^

 Homelessness prevention should be a collaborative effort involving all levels of government and various institutions beyond the homelessness sector. Structural and systems prevention need active participation from mainstream systems, while prevention methods within the homelessness sector, such as early intervention, housing stabilization and eviction prevention, work best when integrated with these broader systems ^24^

 3. Previous research has been acknowledged in the discussion section.

 The first level of the Social Ecological Model highlights how individual demographics and economic status play a crucial role in homelessness. It shows that many homeless individuals in Malaysia struggle with insufficient income, inability to afford housing, and a lack of skills. Previous studies have identified economic instability and rising living costs as significant contributors to the growing homeless population in the country. ^5^ These findings are further supported by ^5,10^ linking homelessness to factors such as unemployment, low income, inability to secure proper housing, lack of affordable housing, and limited access to transportation for work . ^7,10^ Compounding these challenges, many homeless individuals experience a sense of abandonment due to fractured family relationships ^8^. The study also revealed that homeless respondents lacked the essential skills needed to obtain stable employment. This observation aligns with the research of ^14,15^ which demonstrated that developing occupational skills can significantly enhance the well-being and prospects of those facing unemployment and homelessness. The persistent lack of education, training, and life skills continues to be a major barrier to employment ^13,32^

 The second level of the Social Ecological Model emphasizes that homeless individuals frequently suffer from weak interpersonal relationships with family, peers, and social networks. Many respondents reported experiencing family conflicts and strained ties with relatives and friends. This observation is consistent with previous studies ^5,7,9^ which indicate that homeless individuals often feel abandoned, endure social isolation, and lack sufficient social support and resources. It was revealed that homeless individuals suffer from deficiencies in familial and community support, as well as limited interactions with service providers ^9^.  Nevertheless, the finding revealed that some respondents expressed confidence in their ability to overcome their current situation. The finding is consistent with ^17^ who found that the homeless who often viewed as a burden on society due to their nonconformity to social norms, can transform themselves when given proper training and rehabilitation, as they possess the ability to plan for a better future. However, these efforts are unlikely to succeed if the homeless lack the determination to resolve their issues, causing them to revert to their old ways, even after receiving support ^16^. ^18^ discovered that about eight out of 45, returned to the streets despite undergoing rehabilitation programs.

 The third level focuses on the relationship between homeless individuals and the broader community, including associations and informal networks, revealing that they often feel insecure and marginalized from societal participation due to their circumstances. This finding aligns with the study by ^9^ who observed that homeless individuals frequently experience isolation, remain silent, and perceive discrimination because of their situation ^.^ Similarly, it was noted that perceived estrangement and social exclusion can exacerbate societal divides, diminishing social trust and cohesion, and leaving individuals feeling disconnected and unable to engage in meaningful activities ^20,21^

 The fourth level of the model focuses on the role of organizations in impacting the lives of homeless individuals. These organizations provide essential support, such as food, monetary assistance, and shelter. This finding aligns with ^22,23^ who emphasized that homeless individuals require physical support in the form of shelter, food, and medical care, as well as societal interventions to address fundamental human needs. Supportive relationships with caregivers and targeted programs that integrate care and services are crucial approaches to consider in effectively assisting the homeless.

 The fifth level is policy which comprises local, state, and national policies for citizens. The respondents claimed that the current governmental policies do not favour them and should not interfere with their life. The findings align with the study conducted by ^25 ^stated that the authorities under Destitute Persons Act 1977 (DPA 1977) can forcibly remove individuals they consider homeless and place them in *Desa Bina Diri (DBD),* a shelter that offers protection with the court order. DPA 1977 has faced significant criticism for violating personal liberty and freedom of movement of person who is categorized as a destitute person. Nonetheless, the finding also show that some respondents viewed these government agencies as friendly and supportive. The finding corroborates with ^25,33  ^ who revealed that some homeless individuals, particularly senior citizens, were rescued from their difficult lives on the streets by the authorities.  They were given necessary assistance including shelter. Nevertheless, the concept of 'rehabilitation' must be critically examined to determine whether it genuinely addresses the needs of those experiencing homelessness ^25 ^The policies should focus on establishing a robust support system for the homeless, while also empowering the elderly and strengthening their communities ^33^.

 4. The conclusion has been updated accordingly.

 To conclude, eradicating urban homelessness requires a comprehensive approach rooted in the five pillars of the structural ecological model. This study underscores the importance of equipping homeless individuals with employment skills (individual), fostering peer support (interpersonal), promoting social acceptance (community), ensuring access to rehabilitation services (organizational), and implementing a coordinated governmental strategy (policy). While current efforts provide essential support like health services, temporary shelters, and food, they often fail to address the other root causes of homelessness, such as unemployment, loss of income, lack of skills and social support.  Therefore, it is imperative for the government to intensify efforts by funding programs that enhance literacy and provide Technical and Vocational Education and Training (TVET). These programs should offer formal, non-formal, and informal learning opportunities that may collaborate with communities and organizations that empower individuals to secure meaningful employment and rebuild their lives. Additionally, the government shall prioritize accessible housing for the homeless, similar to the " *Projek Perumahan Rakyat Termiskin (PPRT)*" or Housing Project for the Absolute Poor, ensuring that these projects are widespread and located near essential amenities. Providing permanent housing to meet the basic needs of the homeless can significantly alleviate their hardships and improve their overall well-being. By providing stable housing, communities, and organizations can further support the homeless by offering employment opportunities, with the government incentivizing employers to participate. This integrated approach is vital to enabling homeless individuals to reintegrate into society and sustain long-term stability.

 A comprehensive and well-structured support system is essential to effectively eradicate urban homelessness in the country. This system should be designed to address the root causes of homelessness, such as poverty, lack of affordable housing, unemployment and lack of social support. It would involve coordinated efforts from government agencies, non-governmental organizations, and the private sector, focusing on providing not only immediate relief but also long-term solutions. These solutions could include affordable housing programs, job training and employment opportunities, and social support networks. By integrating these components into a cohesive and sustainable framework, the desired support system would not only provide immediate assistance to those experiencing homelessness but also work towards preventing future homelessness, ultimately leading to the complete eradication of urban homelessness in the country. Future research could investigate the effectiveness of specific support system components, such as job training or affordable housing programs, in preventing homelessness. Additionally, it could focus on assessing the long-term sustainability and scalability of these solutions across different urban areas to optimize and adapt the support system for eradicating homelessness.

# Reviewer report: Reviewer response for version 1

**DOI:** 10.5256/f1000research.77191.r119654

**Published:** 2022-02-07

**Authors:** Zakiyah Jamaluddin

**Affiliations:** 1 School of Applied Psychology, Social Work & Policy, Universiti Utara Malaysia (UUM), Kuala Lumpur, Malaysia

1. The method section should include the following details :

 a. sampling technique - how were all 65 respondents chosen? is it based on purposive or convenience sampling?

 b. face to face interview - is it a structured interview using questionnaires, or a semi structured/unstructured interview using protocol interview?

 2. Table 2 should be in Findings.

 3. Discussion - Should be related to the literature review and how it relates to the title of this manuscript.

 4. Conclusion - Should be related to the findings and discussion but there was little discussion in the article, only highlighting of results instead.

Is the work clearly and accurately presented and does it cite the current literature?

Yes

If applicable, is the statistical analysis and its interpretation appropriate?

Yes

Are all the source data underlying the results available to ensure full reproducibility?

Partly

Is the study design appropriate and is the work technically sound?

Yes

Are the conclusions drawn adequately supported by the results?

Partly

Are sufficient details of methods and analysis provided to allow replication by others?

Partly

Reviewer Expertise:

Youth and Community

I confirm that I have read this submission and believe that I have an appropriate level of expertise to confirm that it is of an acceptable scientific standard, however I have significant reservations, as outlined above.

# Response

**Published:** 2024-08-31

**Authors:** Noor Ashikin Mohd Rom

**Affiliations:** Multimedia University, Malaysia

1. Method Section

 a. Convenience sampling was applied in this study, as this method involves selecting individuals who are readily available and willing to participate. The study employed a social ecological Model to survey sixty-five (65) homeless people. The survey respondents were identified by *Pertubuhan Kebajikan ArRiqab*, one of the active non-governmental organisations (NGOs) that assisting homeless people in Kuala Lumpur area  A simple regression analysis requires a minimum of 50 samples and sample-to-variable ratio suggests a minimum observation-to-variable ratio of 5:1. ^30^ Additionally, minimum sample size of N ≥ 50+m (where m is the number of predictors) was recommended. ^31^ The minimum sample size by applying ^31 ^method is 55, however, the researchers managed to get 65 respondents.

 b. This is a structured interview using questionnaires.

 2. Table 2 has been included in Findings.

 3. Discussion has been updated accordingly that related to literature review and title.

 The first level of the Social Ecological Model highlights how individual demographics and economic status play a crucial role in homelessness. It shows that many homeless individuals in Malaysia struggle with insufficient income, inability to afford housing, and a lack of skills. Previous studies have identified economic instability and rising living costs as significant contributors to the growing homeless population in the country. ^5^ These findings are further supported by ^5,10^ linking homelessness to factors such as unemployment, low income, inability to secure proper housing, lack of affordable housing, and limited access to transportation for work . ^7,10^ Compounding these challenges, many homeless individuals experience a sense of abandonment due to fractured family relationships ^8^. The study also revealed that homeless respondents lacked the essential skills needed to obtain stable employment. This observation aligns with the research of ^14,15^ which demonstrated that developing occupational skills can significantly enhance the well-being and prospects of those facing unemployment and homelessness. The persistent lack of education, training, and life skills continues to be a major barrier to employment ^13,32^

 The second level of the Social Ecological Model emphasizes that homeless individuals frequently suffer from weak interpersonal relationships with family, peers, and social networks. Many respondents reported experiencing family conflicts and strained ties with relatives and friends. This observation is consistent with previous studies ^5,7,9^ which indicate that homeless individuals often feel abandoned, endure social isolation, and lack sufficient social support and resources. It was revealed that homeless individuals suffer from deficiencies in familial and community support, as well as limited interactions with service providers ^9^.  Nevertheless, the finding revealed that some respondents expressed confidence in their ability to overcome their current situation. The finding is consistent with ^17^ who found that the homeless who often viewed as a burden on society due to their nonconformity to social norms, can transform themselves when given proper training and rehabilitation, as they possess the ability to plan for a better future. However, these efforts are unlikely to succeed if the homeless lack the determination to resolve their issues, causing them to revert to their old ways, even after receiving support ^16^. ^18^ discovered that about eight out of 45, returned to the streets despite undergoing rehabilitation programs.

 The third level focuses on the relationship between homeless individuals and the broader community, including associations and informal networks, revealing that they often feel insecure and marginalized from societal participation due to their circumstances. This finding aligns with the study by ^9^ who observed that homeless individuals frequently experience isolation, remain silent, and perceive discrimination because of their situation ^.^ Similarly, it was noted that perceived estrangement and social exclusion can exacerbate societal divides, diminishing social trust and cohesion, and leaving individuals feeling disconnected and unable to engage in meaningful activities ^20,21^

 The fourth level of the model focuses on the role of organizations in impacting the lives of homeless individuals. These organizations provide essential support, such as food, monetary assistance, and shelter. This finding aligns with ^22,23^ who emphasized that homeless individuals require physical support in the form of shelter, food, and medical care, as well as societal interventions to address fundamental human needs. Supportive relationships with caregivers and targeted programs that integrate care and services are crucial approaches to consider in effectively assisting the homeless.

 The fifth level is policy which comprises local, state, and national policies for citizens. The respondents claimed that the current governmental policies do not favour them and should not interfere with their life. The findings align with the study conducted by ^25 ^stated that the authorities under Destitute Persons Act 1977 (DPA 1977) can forcibly remove individuals they consider homeless and place them in *Desa Bina Diri (DBD),* a shelter that offers protection with the court order. DPA 1977 has faced significant criticism for violating personal liberty and freedom of movement of person who is categorized as a destitute person. Nonetheless, the finding also show that some respondents viewed these government agencies as friendly and supportive. The finding corroborates with ^25,33  ^ who revealed that some homeless individuals, particularly senior citizens, were rescued from their difficult lives on the streets by the authorities.  They were given necessary assistance including shelter. Nevertheless, the concept of 'rehabilitation' must be critically examined to determine whether it genuinely addresses the needs of those experiencing homelessness ^25 ^The policies should focus on establishing a robust support system for the homeless, while also empowering the elderly and strengthening their communities ^33^ .

 4. Conclusion has been updated accordingly. 

 To conclude, eradicating urban homelessness requires a comprehensive approach rooted in the five pillars of the structural ecological model. This study underscores the importance of equipping homeless individuals with employment skills (individual), fostering peer support (interpersonal), promoting social acceptance (community), ensuring access to rehabilitation services (organizational), and implementing a coordinated governmental strategy (policy). While current efforts provide essential support like health services, temporary shelters, and food, they often fail to address the other root causes of homelessness, such as unemployment, loss of income, lack of skills and social support.  Therefore, it is imperative for the government to intensify efforts by funding programs that enhance literacy and provide Technical and Vocational Education and Training (TVET). These programs should offer formal, non-formal, and informal learning opportunities that may collaborate with communities and organizations that empower individuals to secure meaningful employment and rebuild their lives. Additionally, the government shall prioritize accessible housing for the homeless, similar to the "Projek Perumahan Rakyat Termiskin (PPRT)" or Housing Project for the Absolute Poor, ensuring that these projects are widespread and located near essential amenities. Providing permanent housing to meet the basic needs of the homeless can significantly alleviate their hardships and improve their overall well-being. By providing stable housing, communities, and organizations can further support the homeless by offering employment opportunities, with the government incentivizing employers to participate. This integrated approach is vital to enabling homeless individuals to reintegrate into society and sustain long-term stability.

 A comprehensive and well-structured support system is essential to effectively eradicate urban homelessness in the country. This system should be designed to address the root causes of homelessness, such as poverty, lack of affordable housing, unemployment and lack of social support. It would involve coordinated efforts from government agencies, non-governmental organizations, and the private sector, focusing on providing not only immediate relief but also long-term solutions. These solutions could include affordable housing programs, job training and employment opportunities, and social support networks. By integrating these components into a cohesive and sustainable framework, the desired support system would not only provide immediate assistance to those experiencing homelessness but also work towards preventing future homelessness, ultimately leading to the complete eradication of urban homelessness in the country. Future research could investigate the effectiveness of specific support system components, such as job training or affordable housing programs, in preventing homelessness. Additionally, it could focus on assessing the long-term sustainability and scalability of these solutions across different urban areas to optimize and adapt the support system for eradicating homelessness.
